# A design-by-treatment interaction model for network meta-analysis with random inconsistency effects

**DOI:** 10.1002/sim.6188

**Published:** 2014-04-29

**Authors:** Dan Jackson, Jessica K Barrett, Stephen Rice, Ian R White, Julian PT Higgins

**Affiliations:** a MRC Biostatistics UnitCambridge, U.K.; b Centre for Reviews and Dissemination, University of YorkU.K.; c University of BristolU.K.

**Keywords:** inconsistency, mixed treatment comparisons, multiple treatments meta-analysis, network meta-analysis, sensitivity analysis

## Abstract

Network meta-analysis is becoming more popular as a way to analyse multiple treatments simultaneously and, in the right circumstances, rank treatments. A difficulty in practice is the possibility of ‘inconsistency’ or ‘incoherence’, where direct evidence and indirect evidence are not in agreement. Here, we develop a random-effects implementation of the recently proposed design-by-treatment interaction model, using these random effects to model inconsistency and estimate the parameters of primary interest. Our proposal is a generalisation of the model proposed by Lumley and allows trials with three or more arms to be included in the analysis. Our methods also facilitate the ranking of treatments under inconsistency. We derive *R* and *I*^2^ statistics to quantify the impact of the between-study heterogeneity and the inconsistency. We apply our model to two examples. © 2014 The Authors. Statistics in Medicine published by John Wiley & Sons, Ltd.

## 1. Introduction

Meta-analysis is well established in medical statistics. Typically, meta-analyses involve pairwise comparisons of two treatments, but, because it is usually important to consider more than two treatment alternatives when making decisions in practice, network meta-analysis has attracted much interest in recent years. In network meta-analysis, multiple treatments are included in the meta-analysis, using data from trials that compare at least two of these treatments. Network meta-analysis offers the advantage of being able to compare any treatments included in the network, including those that have not been compared directly, and, in the right circumstances, allows the full set of treatments to be ranked. However, all the usual issues surrounding the application of meta-analysis apply in the network meta-analysis setting and may even be exacerbated when more than two treatments are analysed simultaneously 1. Furthermore, the more complicated statistical modelling required in network meta-analysis presents problems. For example, Song *et al.* 2 identify a variety of methodological problems associated with network meta-analysis, including ‘an unclear understanding of underlying assumptions’, the ‘use of inappropriate or flawed methods’ and ‘inadequate comparison or inappropriate combination of direct and indirect evidence’.

Perhaps, the biggest statistical challenge facing network meta-analysis is a problem that has been called inconsistency or incoherence 3–7. This arises when treatment effects obtained from direct evidence and indirect evidence (or from two different types of indirect evidence) do not agree. Inconsistency is problematic statistically, because models must incorporate it and, conceptually, because it is less clear what estimates of treatment effect mean when the sources of evidence are observed to be inconsistent. Methods for network meta-analysis began by distinguishing between direct and indirect sources of evidence and then combining across them 8. This led naturally to the idea of ‘node splitting’ 9–11, where inferences are split depending on whether the information comes from studies that provide direct or indirect information about a particular effect. Node splitting aims to explore in detail where inconsistency may lie, and should be conducted after a global assessment of inconsistency. Here, we adopt an alternative modelling approach, where instead of splitting nodes, we introduce a further source of variation to allow for inconsistency in the network. A case for treating the inconsistency parameters as fixed effects (separate parameters that are assumed to be unrelated) is made by Higgins *et al*. 7 who argue that the common distribution assumption implicit in the random-effects formulation is implausible. This is, they argue, because ‘each inconsistency parameter has its own interpretation and some may be a priori more likely to be nonzero than others’. One important practical advantage of using fixed effects for the inconsistency parameters is that the resulting model can be fitted as a multivariate meta-regression 12. However, using fixed effects for the inconsistency parameters makes ranking treatments across all designs a challenging task, and sensitivity analyses involve using multiple sensitivity parameters to describe the inconsistency.

In contrast, the approach we adopt here is to introduce inconsistency parameters into the model as random effects, so that the inconsistency across evidence sources is conceptualised as additional variation, in the same way as heterogeneity across studies is conceptualised as variation in a conventional random-effects meta-analysis 13. Modelling the inconsistency parameters as following a common (random-effects) distribution is at best a strong assumption and at worst a wrong one. However, it offers a useful modelling framework because it facilitates the ranking of treatments under inconsistency and sensitivity analyses in terms of a single sensitivity parameter (the inconsistency variance). Perhaps most importantly, our random-effects formulation of the design-by-treatment interaction model enables us to estimate average treatment effects across all designs, rather than the design-specific treatments effects we obtain when using fixed effects for the inconsistency parameters 12. We return to the case for and against using random effects for the inconsistency parameters in the discussion.

Our approach to modelling network meta-analytic data is motivated by the recently proposed design-by-treatment interaction model 7,12 and by the model proposed by Lumley 6 for the network meta-analysis of two-arm trials with normally distributed data. Our model is a special case of the design-by-treatment interaction model and a generalisation of Lumley's model. It is also closely related to models for loop inconsistency, which assume that the inconsistency parameters are normally distributed 5; as shown by Higgins *et al.* 7, loop inconsistency models contain a subset of the inconsistency parameters of the design-by-treatment interaction model, so some reduced forms of our model are loop inconsistency models. Hence, if the inconsistency is genuinely due to loop inconsistency, then using the design-by-treatment interaction model, rather than the loop inconsistency model, incurs a loss of power, and other issues with over-parameterisation could arise. We propose a new model for network meta-analysis, but we draw inspiration from a variety of different models and approaches. As another innovation, we also take the recently proposed multivariate heterogeneity statistics 14 and show how these can be used to quantify the impact of heterogeneity and inconsistency.

Useful properties of our model are its abilities to incorporate trials with three or more arms, to use exact distributions and to incorporate covariates. The ability to include clinically relevant covariates is especially useful because these could be used to explain the inconsistency, an important point to which we return in the discussion. We assume that attempts have been made to account for inconsistency when presenting our model, where these attempts could include strategies such as introducing covariates or removing unsatisfactory studies. The rest of the paper is set out as follows. In section 2, we develop our model and show how it is related to some established models. In section 3, we show how this model may be estimated and used both in the context of a fully estimation-based procedure and also in the context of a sensitivity analysis. In section 4, we derive statistics to quantify the impact of the variance components in our model, and we apply our methods to two example datasets in section 5. We conclude with a discussion in section 6.

## 2. The model

Our model is intended for network meta-analysis, where multiple studies make different comparisons from a set of treatments and some studies may have more than two treatment arms. It is possible to fit models for network meta-analysis using either contrast-based or arm-based analyses 3. In an arm-based analysis, the outcome data are the studies’ treatment arm outcomes, such as mean values. In a contrast-based analysis, the outcome data are estimated treatment effects, such as mean differences. Our proposed model is a special case of the recently developed design-by-treatment interaction model 7,12, which focusses on the treatment effects. Hence, the model development that follows is most naturally conceptualised in terms of a contrast-based analysis. Without loss of generality, we take treatment *A* as the reference treatment and refer to the other treatments as *B*, *C*, *D* and so on. When referring to the design-by-treatment interaction model, we take the design *d* as referring only to the set of treatments compared in a trial 7,12. For example, if the first design compares treatments *A* and *B* only, then *d* = 1 refers to two-arm trials that compare these two treatments.

### 2.1. The design-by-treatment interaction model

The design-by-treatment interaction model has recently been proposed by Higgins *et al.* 7 and has been implemented using fixed effects for the inconsistency parameters by White *et al.* 12. This model is defined in terms of the treatment effects or ‘contrasts’. See Higgins *et al.* 7 for a full explanation of why the design-by-treatment interaction model successfully addresses the complications that arise from the presence of multi-arm trials in the models proposed by Lu and Ades 5. In brief, the inconsistency parameters included in loop inconsistency models depend on the ordering of the treatments: ‘The only model that contains all the Lu–Ades models (i.e. with all different treatment orderings) appears to be the design-by-treatment interaction model’ 7.

The design-by-treatment interaction model can be viewed in two ways. From the first viewpoint, we model the contrasts of all treatments including those not used in particular designs. Thus, for example, we include a model for the *C* − *D* contrast in trials that compare treatments *A* and *B* only. These contrasts are to be thought of as counterfactual contrasts that would have been observed if these trials had, counter to fact, also included treatments *C* and *D*. Using counterfactual contrasts provides a particularly simple model form. However, from the second viewpoint, the model can be written to describe only the contrasts needed in application. These two viewpoints lead to the same likelihood, and the assumptions about counterfactual contrasts in the first viewpoint are not used in estimation. We use the first viewpoint in sections 2.1– 2.3 and the second viewpoint in sections 2.4– 2.6.

From the first viewpoint, we provide a model for all contrasts 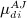, where 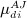 denotes the true treatment effect of treatment *J* relative to the reference treatment *A* in the *i*th study of design *d*. We assume consistency within designs, so 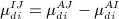. Before presenting the design-by-treatment interaction model, we will discuss some special cases of this, in increasing order of complexity, in order to describe the various components that comprise our model. In our framework, the common-effect and consistent model assumes that

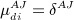


where *δ*^*AJ*^ is the effect of treatment *J* relative to treatment *A*. The common-effect and consistent model therefore assumes that all studies of all designs have the same true treatment effects. The *δ*^*AJ*^ are *c* parameters of primary interest, which are referred to as the basic parameters 7, where *c* equals the total number of treatments in the network minus one. These basic parameters describe the effectiveness of treatments relative to treatment *A*. The contrasts 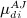 could represent differences in mean responses, log-odds ratios or any other measure of treatment effect used in meta-analysis.

The random-effects and consistent model assumes that

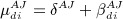


where 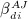 is a study-by-treatment interaction term to reflect between-study heterogeneity. It is conventional to assume that the 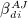 are normally distributed, centred at zero, where the variances of 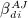, with suitable covariances defined later in the text, provide the between-study variance structure.

Finally, the design-by-treatment interaction model assumes that

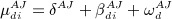
1 where 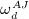 is a design-by-treatment interaction term to reflect inconsistency (variability between designs). The 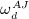, which we refer to as the inconsistency parameters, allow inconsistency within the network, but if all 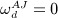, then the consistency assumption is satisfied. Equation (1) is given as Equation (3) by Higgins *et al.* 7. The design-by-treatment interaction model implies that

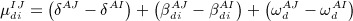
2 where 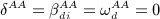, so that Equation (4) simplifies to Equation (3) when *I* = *A*. Hence, model (4), with these constraints, describes the design-by-treatment interaction model. Equation (4) is a statement concerning the assumptions made about the studies’ true treatment effects and does not incorporate within-study sampling variation. Further within-study distributional assumptions are also needed to specify the likelihood, as we explain in section 2.6.

The 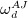 terms mean that different contrasts for each treatment comparison are allowed for every design, and these terms model the inconsistency in the network 7. This allows, for example, for the average contrasts in trials involving treatments *A* and *B* only to be different to those in trials involving treatments *A*,* B* and *C*. This is often plausible. For example, a treatment might be investigated and discounted by earlier trials, so that the trials comparing treatments *A* and *B* only would tend to be performed more recently. Differences in study practices and patient populations over time could then also result in different contrasts between the two designs. However, we do assume that the consistency equations apply to the average treatment effects across all designs and studies.

### 2.2. Modelling the between-study heterogeneity

In order to apply the design-by-treatment interaction model, we must specify the distributional assumptions made about the form of the heterogeneity and the inconsistency parameters. We model the heterogeneity using one of the simplest models that has been proposed

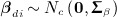
3 where ***Σ***_*β*_ denotes a square matrix where the entries on the leading diagonal are 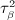 and all other entries are 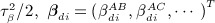 and *N*_*c*_ denotes a multivariate normal distribution in *c* dimensions. This form of ***Σ***_*β*_ is justified by the assumption that the heterogeneity variance is the same for all treatment comparisons for every study 3. We do, however, agree with Salanti *et al.* 3 that this assumption ‘may sometimes be difficult to defend’, and so, we return to the possibility of alternative, and more general, models for the heterogeneity in the discussion.

### 2.3. Modelling the inconsistency parameters

Our proposed model for the inconsistency parameters is an innovation of our proposed methodology. As explained in the introduction, we use random effects for the inconsistency parameters 5,6, and we assume that

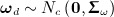
4 where 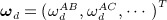 and ***Σ***_*ω*_ denotes a square matrix where the entries on the leading diagonal are 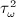 and all other entries are 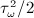. This form of ***Σ***_*ω*_ is justified in a very similar way to the assumed form of ***Σ***_*β*_ in the previous subsection; we assume that the inconsistency variance across designs is the same from all treatment comparisons. The inconsistency variance 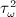 quantifies the extent of the inconsistency in the network as a whole, and specific inconsistency parameters describe where particular inconsistencies arise. If 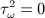, then there is no inconsistency, and the model simplifies to a consistency model.

The 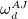 are of interest because their estimates can be used to explore any inconsistencies in the network. These inconsistencies occur where treatment effects in particular designs differ from the average across all designs. In order to explain why inconsistencies occur, the model alone is not enough, and detailed knowledge of the design and conduct of the trials, including their risk of bias, is crucial. The model using fixed effects for the inconsistency parameters proposed by White *et al.* 12 can also be used to explore inconsistencies and may be considered preferable to the proposed model in situations where identifying inconsistencies is the primary aim. This is because the approach of White *et al.* does not make the strong assumption that the 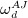 are normally distributed, so that inferences cannot be driven by this distributional assumption. A complication, however, is that the interpretation of fixed-effect inconsistency terms depends on the parameterisation used 12. This is *not* the case here because we place exchangeable inconsistency parameters on all treatments in all designs.

Together, Equations (5) and (6) provide a random-effects model that incorporates both between-study heterogeneity and inconsistency, where the extent of these two sources of variation is described by the parameters 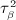 and 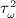, respectively. The true effects from separate studies of the same design are not independent because they share the same inconsistency parameter. However, the true effects from trials of different designs are independent. We ignore any possible structure in the inconsistency parameters; for example, in some situations, it may be plausible that particular inconsistency parameters are more alike than others. This possibility is not taken into account when assuming that the inconsistency parameters are exchangeable.

We interpret the basic parameters *δ*^*AJ*^ as representing the average treatment effects across all studies and designs. An advantage of our framework is that it can easily be extended to incorporate more complicated forms of ***Σ***_*β*_ and ***Σ***_*ω*_ in situations where these are feasible. We return to this issue in the discussion.

### 2.4. Using the model to describe network meta-analysis datasets with two-arm trials

As explained earlier, the design-by-treatment interaction model (4) describes counterfactual contrasts, so we now take only those needed in application and hence adopt the second viewpoint. If all studies are two-arm trials, then we only need a single contrast from model (4) for each trial. Let *δ*_*d*_ denote *δ*^*AJ*^ − *δ*^*AI*^ where *I* and *J* are the arms that are included in design *d*. Then the contrast from model (4) used for the *i*th trial of design *d* is *δ*_*d*_ + *B*_*di*_ + *W*_*d*_, where 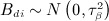 and 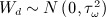. If 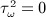 and normal within-study distributions are assumed, then this is a conventional model that assumes consistency and can be fitted as a standard intercept-free meta-regression model 12.

More generally, but continuing to assume normal within-study distributions, our model is a reparameterisation of the model proposed by Lumley 6, p. 2317, where our 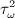 and 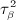 are denoted as *ω*^2^ and 2*τ*^2^ by Lumley. Like us, Lumley uses a contrast-based analysis, but he uses two random heterogeneity terms, which are presumably intended to reflect the presence of heterogeneity in the two treatment arms. These two heterogeneity terms can be absorbed into a single random effect for the contrast, where this random effect need not result from equal heterogeneity variances in both arms.

### 2.5. Using the model to describe network meta-analysis datasets where trials have more than two arms

Let *c*_*d*_ denote the number of linearly independent contrasts estimable from design *d*, so that *c*_*d*_ equals the number of treatment arms in design *d* minus one. In order to take a subset of *c*_*d*_ contrasts from 4 that conveniently describe the data, for each design, we take a ‘baseline’ treatment arm *B*_*d*_. We then compute the effects of the other treatments relative to the baseline treatment arm. Other contrasts also estimable from design *d* can be obtained as linear combinations of this subset of contrasts 3. Hence, these contrasts are sufficient, and the same likelihood function is obtained regardless of the baseline groups used. Thus, we use the *c*_*d*_ contrasts from design *d*

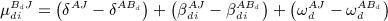
5 where the 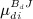 in Equation (7) are a subset of the contrasts from 4, where the *J* are restricted to the *c*_*d*_ ‘non-baseline’ treatments included in design *d*.

To model the data, we need the distributions of the heterogeneity and inconsistency components in (7). Let 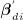 denote the subvector of ***β***_*di*_, of length *c*_*d*_, containing the 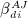 described in (7). Assumption (5) and the assumed form of ***Σ***_*β*_ means that

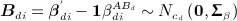
6 where ***1*** is the vector of length *c*_*d*_ where every entry is one. Similarly, let 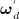 denote the subvector of ***ω***_*d*_, of length *c*_*d*_, containing the 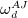 from (7). Assumption (6) and the assumed form of ***Σ***_*ω*_ means that

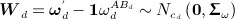
7 so that 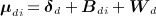
8 where ***μ***_*di*_ is the vector of length *c*_*d*_ containing the 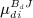 from (7) and ***δ***_*d*_ is the vector of length *c*_*d*_ containing the corresponding 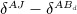 also from (7). We define 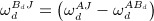 so that these terms are the entries of ***W***_*d*_. Equation (10), with the distributional assumptions in 8 and 9, models all contrasts in the data and so provides our basis. Equations 8 and 9 describe the between-study heterogeneity and the inconsistency, respectively.

### 2.6. The proposed model for normally distributed and binary data

Equations 8, 9 and (10) only describe the true treatment effects, and further within-study distributional assumptions are needed to model outcome data. For multivariate normal outcome data from multi-arm studies, our model for the treatment effects in a contrast-based analysis is

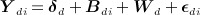
9 where ***Y***_*di*_ is the vector of estimated treatment effects and ***ε***_*di*_ is multivariate normal with within-study covariance matrix ***S***_*di*_. Because the estimates of treatment effect share a common baseline treatment group, the within-study covariances (the non-diagonal entries of ***S***_*di*_) are nonzero, and it is important that these are not ignored in the analysis 15. Model 11 is a multivariate extension of Lumley's model 6 and can also be thought of as a generalisation of a particular type of loop inconsistency model, because the design-by-treatment interaction model contains additional inconsistency parameters to loop inconsistency models 7. For normally distributed data, or where a normal approximation is used for other types of data, model 11 is assumed in a contrast-based analysis.

However, and with a little extra effort, the design-by-treatment interaction model can also be applied in the context of an arm-based analysis. Here, we further use study-specific fixed effects for the arm-level parameters in the baseline treatment arms or for the average arm-level parameter across all treatment arms. The arm-level parameters in the other trial arms are then defined using the appropriate contrasts from 10. Upon making an appropriate distributional assumption, the observed data in all trial arms are modelled using these arm-level parameters. Arm-based analyses therefore facilitate using exact distributions in the likelihood for non-continuous data. For example, for binary data and a logistic link function, we assume that the number of events in treatment arm 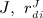, is distributed as

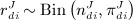


where 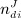 is the number of patients in this treatment arm and

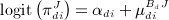


where *α*_*di*_ denotes the log odds of an event in the baseline treatment arm in the *i*th study of design *d*.

## 3. Estimation

We perform both contrast-based and arm-based analyses in section 5. Only the treatment effects are available to us for all studies in our first example (i.e. not all arm-level data are available), so here, we use a contrast-based analysis. This example involves continuous outcome data, so the direct use of model 11, which assumes multivariate normality, is perfectly natural. However, we use an arm-based analysis for our second example, where the necessary arm-level binary outcome data are available, so that binomial distributions can be used directly. We use WinBUGS 16 as a convenient tool for fitting our model because it is quite a complicated mixed, and possibly generalised, linear model. WinBUGS is therefore a natural environment for fitting models of this type, but our use of WinBUGS to fit Bayesian models comes at the price of issues surrounding prior sensitivity and checking convergence diagnostics.

By adopting a Bayesian approach, the probabilistic ranking of the treatments follows more naturally than in a classical framework 12. To rank the treatments, at each iteration of the Markov chain Monte Carlo (MCMC), we record which treatment is most effective, that is, which basic parameter is the largest. If all basic parameters are of the same sign and indicate that the reference treatment *A* is most effective, then we take treatment *A* as most effective. The proportion of MCMC iterations in which treatments are the most effective gives the probabilities that they are most effective. These probabilities can then be used to rank the treatments. The supplementary material contains sections of WinBUGS code, described as ‘probabilities of being the best treatment’, which show the calculations used when performing this ranking. This method for ranking the treatments could be extended by calculating the probabilities that each treatment is the second most effective, the third most effective and so on, so that the surface under the cumulative ranking (SUCRA) line for each treatment can be calculated 17. By using the basic parameters to rank the studies in this way, the ranking is an average across designs, and design-specific rankings may differ. By modelling the inconsistency parameters as random effects, we are able to perform ranking without making the assumption of consistency, but we return to this issue in the discussion.

Our arm-based analyses using WinBUGS are similar to other Bayesian meta-analyses 18,19 in many respects. We give all location parameters vague (normal with very small precision) prior distributions, and uniform prior distributions from 0 to 5 were used for *τ*_*β*_ and *τ*_*ω*_. Prior sensitivity to apparently vague priors for the between-study variance can be considerable in univariate random-effects meta-analysis 20, and this can also be anticipated to be the case for the variance structure in more complex models such as ours. Hence, our choice of prior distributions should be taken as providing some indicative results. Very large burn-ins of 30 000 were used to ensure convergence, which was checked by running three chains at different starting values and using WinBUGS’ implementation of the Gelman–Rubin convergence statistic, as modified by Brooks and Gelman 21, which were stable in all instances. For inference, enough iterations were used to ensure that all Monte Carlo standard errors were around 0.005 (the maximum for all parameters monitored was 0.0069). We are therefore confident in quoting results to two decimal places. Our WinBUGS code, which has been adapted from code provided by Dias *et al.* 10,22,23, is provided in the supporting information that accompanies the paper. An alternative to using WinBUGS for fitting the proposed model would be to develop a computer program for fitting models of this kind, perhaps using maximum likelihood or restricted maximum likelihood. Purpose built programs and packages using classical methods to fit meta-analytic models with specific forms of random-effects structures have been developed 24–26. Because our model is a concrete proposal for a model for network meta-analysis, developing suitable computer software for fitting it routinely could be an important next step; the ranking of the treatments could be performed in a classical framework using the type of procedure proposed by White *et al.* 12.

An alternative to estimating the full model is to perform a sensitivity analysis using 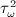 as the sensitivity parameter. If 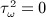, then all inconsistency parameters are zero, and the model reduces to a model that assumes consistency, as commonly assumed in practice 2. However, as 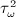 increases, we can determine how the inferences depend on the assumed extent of the inconsistency. This type of sensitivity analysis is likely to be especially useful in situations with limited data relative to the number of parameters to estimate. This is because, in such situations, data provide little information about the extent of the inconsistency so that the resulting estimation of the full model would be very imprecise. We illustrate this idea using our second example in section 5.

## 4. *R* and *I*^2^ statistics for the impact of between-study heterogeneity and inconsistency

Multivariate *R* and *I*^2^ statistics can be computed as explained by Jackson *et al.* 14. A classical approach was used when originally developing these statistics, but here, we adopt Bayesian estimation. Hence, we use the normal approximations to credible regions, whereas Jackson *et al.* used the (approximate) covariance matrices of the estimated effects, but otherwise, the same type of procedure is adopted. In situations where Bayesian and maximum likelihood analyses are in good numerical agreement, *R* and *I*^2^ statistics very similar to those in the succeeding discussions would be obtained when using likelihood-based methods.

In order to quantify the impact of the between-study heterogeneity and inconsistency, we fit three models: the ‘common-effect and consistent’ model (*τ*_*β*_ = *τ*_*ω*_ = 0), the ‘random-effects and consistent’ model (*τ*_*ω*_ = 0) and the ‘random-effects and inconsistent’ (or full) model. We denote the volumes of the normal approximations to the 95% (or any other probability) credible regions for all *c* basic parameters from these three models as *V*_*CC*_,* V*_*RC*_ and *V*_*RI*_, and the covariance matrices of these normal approximations as **C**_*CC*_,* ***C**_*RC*_ and **C**_*RI*_, respectively.

The *R* statistic comparing a model with an additional source (or sources) of variation to an alternative, reduced model is given by the *c*th root of the ratio of the volumes of the credible regions resulting from these two models, where the model with additional variation appears in the numerator.

For example, using the normal approximations to the credible regions, the *R* statistic comparing the ‘random-effects and inconsistent’ and ‘random-effects and consistent’ models is given by

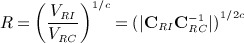


where | ⋅ | denotes the matrix determinant and *I*^2^ = (*R*^2^ − 1) / *R*^2^. These *R* and *I*^2^ statistics quantify the impact of the inconsistency on the random-effects model, where the term ‘impact’ refers to the effect that the inclusion of the extra source of variability (the inconsistency) has on the precision of the estimated treatment effects. These *I*^2^ statistics are interpreted in the same way as conventional *I*^2^ statistics 14 and are subject to the same issues as the conventional univariate one 27. We follow the convention of presenting *I*^2^ statistics as percentages. This type of investigation could be extended by examining particular combinations of treatment effect parameters in the way described by Jackson *et al.* 14, so that the impact of the variance components on some, rather than all, basic parameters may also be quantified. The aforementioned *R* statistic can be interpreted as the factor by which posterior standard deviations are increased on allowing for inconsistency, averaged over all possible treatment contrasts.

As explained by Jackson *et al.* 14, |**C**_*CC*_|^1 / *c*^, |**C**_*RC*_|^1 / *c*^ and | **C**_*RI*_ | ^1 / *c*^ are the squares of geometric means of posterior standard deviations of the basic parameters from the three models. Hence, we calculate the quantities | **C**_*CC*_ | ^1 / 2*c*^,* * | **C**_*RC*_ | ^1 / 2*c*^ and | **C**_*RI*_ | ^1 / 2*c*^ as average posterior standard deviations of the estimated basic parameters resulting from these three models.

Lu and Ades 5 also assess how big the inconsistency is in relation to the heterogeneity. In our notation, they calculate the posterior probability that *τ*_*ω*_ > *τ*_*β*_. We quantify the impact of the inconsistency using our *R* and *I*^2^ statistics and use deviance information criterion (DIC) statistics 5,28 to quantify the strength of evidence that inconsistency is present. Methods for assessing the extent of the heterogeneity and inconsistency based on a generalisation of Cochran's *Q* statistic 14 have also recently been developed for network meta-analysis 29,30. The appropriate use of this type of statistic, in conjunction with the model proposed here, is a possible avenue for further work.

## 5. Applications

In this section, we illustrate our methodology using two contrasting examples. The supplementary material contains the WinBUGS code used for both examples.

### 5.1. Application one: osteoarthritis of the knee

**Figure 1:**
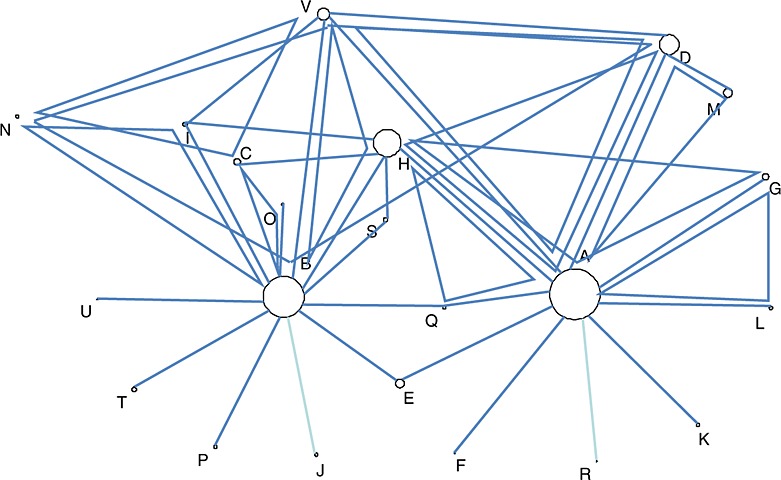
Network diagram for the osteoarthritis of the knee data. Node size is proportional to the number of trials including the treatment, and multi-arm trials are shown as closed loops. Treatments are A: standard care, B: placebo, C: no medication, D: acupuncture, E: balneotherapy, F: braces, G: aerobic exercise, H: muscle exercise, I: heat treatment, J: insoles, K: tai chi, L: weight loss, M: sham acupuncture, N: ice/cooling, O: interferential, P: laser, Q: manual, R: NMES, S: PES, T: PEMF, U: static magnets, V: TENS.

The dataset 31, which has subsequently been updated 32, consists of 87 trials of 38 designs, each comparing a subset of 22 treatments for pain relief for osteoarthritis of the knee (OAK). Most trials contain two arms, except for 10 three-arm trials and one four-arm trial. Outcomes are continuous measurements, and the standardised mean difference of pain at the end of the trial is used to compare treatments, where a negative treatment effect indicates benefit. A network diagram is shown in Figure 1. Note that most of the trials in the network involve comparisons with either standard care or placebo.

Trial arm data are not available for all trials, and we therefore fit the model using a contrast-based approach. For multi-arm trials, the estimates of within-study covariances are not available, so the off-diagonal entries of ***S***_*di*_ are unknown to us. We therefore use an approximate formula for the within-study covariances, given by the reciprocal of the number of patients in the baseline group. This approximation takes the pooled variance *s*^2^ as fixed and common to all treatment arms; denoting the means in each of the three arms as 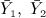 and 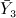, where 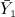 is the mean in the baseline arm common to two standardised mean differences, we take 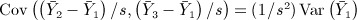, which gives our approximation. For one trial, only treatment contrasts were available, and two arms were in the same treatment category. The two treatment contrasts from this trial were treated as treatment effects from a multi-arm trial with a shared random effect contributing to each treatment effect.

The results from fitting the model are given in Table 1, where we report the means (estimates) and standard deviations of posterior distributions; for variance components we also report posterior medians and 95% credible intervals. We present the two sets of results in Table 1, where we do and do not constrain 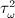 to be zero. By constraining 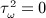, we obtain inferences assuming consistency. By comparing the results, assuming consistency with those from the full model, the implications of relaxing the consistency assumption can be assessed. For all reported results, the posterior medians were similar to posterior means. The posterior mean of the heterogeneity standard deviation *τ*_*β*_ is of the same order of magnitude as the estimated treatment effects. The posterior mean of the inconsistency standard deviation *τ*_*ω*_ in the full model is much smaller, suggesting low levels of inconsistency in the network. Treatment effects suggest that most treatments are more effective than standard care, placebo or no medication, but posterior standard deviations are large because of the sparsity of the evidence in the network. The treatment rankings suggest that interferential treatment is most likely to be the best, followed by static magnets.

**Table 1:** Results for the osteoarthritis of the knee data.

		Consistency 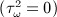		Inconsistency 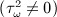	
		Parameter	Estimate (SD)	P(best)	Estimate (SD)	P(best)
Treatment					
A: Standard care	–		0.00		0.00
B: Placebo	*δ*^*AB*^	0.04 (0.21)	0.00	0.04 (0.23)	0.00
C: No medication	*δ*^*AC*^	0.60 (0.32)	0.00	0.59 (0.35)	0.00
D: Acupuncture	*δ*^*AD*^	− 0.78 (0.16)	0.07	− 0.78 (0.19)	0.07
E: Balneotherapy	*δ*^*AE*^	− 0.46 (0.26)	0.00	− 0.49 (0.30)	0.01
F: Braces	*δ*^*AF*^	− 0.15 (0.47)	0.02	− 0.15 (0.50)	0.02
G: Aerobic exercise	*δ*^*AG*^	− 0.60 (0.23)	0.03	− 0.57 (0.25)	0.03
H: Muscle exercise	*δ*^*AH*^	− 0.37 (0.11)	0.00	− 0.36 (0.15)	0.00
I: Heat treatment	*δ*^*AI*^	− 0.03 (0.31)	0.00	− 0.02 (0.33)	0.00
J: Insoles	*δ*^*AJ*^	− 0.01 (0.36)	0.00	0.00 (0.41)	0.00
K: Tai chi	*δ*^*AK*^	− 0.28 (0.29)	0.01	− 0.28 (0.34)	0.01
L: Weight loss	*δ*^*AL*^	− 0.35 (0.26)	0.01	− 0.35 (0.29)	0.01
M: Sham acupuncture	*δ*^*AM*^	− 0.25 (0.24)	0.00	− 0.28 (0.28)	0.00
N: Ice/cooling	*δ*^*AN*^	− 0.25 (0.37)	0.01	− 0.25 (0.39)	0.01
O: Interferential	*δ*^*AO*^	− 1.11 (0.49)	0.51	− 1.11 (0.51)	0.49
P: Laser	*δ*^*AP*^	− 0.24 (0.37)	0.00	− 0.24 (0.42)	0.01
Q: Manual	*δ*^*AQ*^	− 0.29 (0.31)	0.01	− 0.29 (0.32)	0.01
R: NMES	*δ*^*AR*^	0.45 (0.56)	0.00	0.45 (0.58)	0.00
S: PES	*δ*^*AS*^	− 0.70 (0.33)	0.08	− 0.73 (0.36)	0.09
T: PEMF	*δ*^*AT*^	0.01 (0.32)	0.00	0.01 (0.38)	0.00
U: Static magnets	*δ*^*AU*^	− 0.78 (0.58)	0.23	− 0.78 (0.61)	0.23
V: TENS	*δ*^*AV*^	− 0.63 (0.23)	0.01	− 0.61 (0.24)	0.01
					
Heterogeneity mean (SD)	*τ*_*β*_	0.43 (0.06)		0.42 (0.06)	
median (CI)		0.43 (0.32, 0.56)		0.42 (0.30, 0.56)	
Inconsistency mean (SD)	*τ*_*ω*_			0.14 (0.10)	
median (CI)				0.13 (0.00, 0.37)	
					
DIC		132.61		133.23	

NMES: neuromuscular electrical stimulation; PES: pulsed electrical stimulation; PEMF: pulsed electromagnetic fields; TENS:transcutaneous electrical nerve stimulation.

Two sets of results are shown, those assuming consistency and those allowing for inconsistency. Estimates are given by posterior means, and P(best) is the probability that each treatment is best (to two decimal places). Posterior medians and 95% credible intervals are also given for variance parameters.

SD, standard deviation; DIC, deviance information criterion.

Because the inconsistency variance 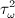 is small, the two sets of results in Table 1 are in good agreement. However, the full model results in (slightly) wider intervals for the estimated treatment effects because it incorporates a further source of variation that is not modelled when assuming consistency.

The *I*^2^ statistics are shown in Table 2. These show that the impact of the inconsistency on the random-effects model is mild (*I*^2^ = 19 *%*), which reinforces our conclusions regarding the magnitude of *τ*_*ω*_ and the small degree of inconsistency, and the DIC statistic for the consistency model is smaller than the one for the inconsistency model. The average standard deviations (section 4) of the estimated treatment effects are 0.15 for the common-effect model assuming consistency, 0.27 for the random-effects model assuming consistency and 0.30 for the random-effects model with inconsistency. The product of the first two *R* statistics shown in Table 2 is equal to the final *R* statistic. This is because the *R* statistics are scaling factors that describe the relative volumes of the uncertainty intervals so this relationship is geometrically obvious. Less obvious, however, is that this relationship also applies to the ‘complement’ of the *I*^2^ statistics; that is, if we define *J*^2^ = (1 − *I*^2^), then these relationships between the *R* statistics also apply to the *J*^2^ (and so, the *J*) statistics. The estimated inconsistency parameters are tabulated in the supplementary materials that accompany the paper.

**Table 2:** *R* and *I*^2^ statistics for the osteoarthritis of the knee data.

	*R*	*I*^2^ (%)
Common-effect and consistent → random-effects and consistent	1.81	69
Random-effects and consistent → random-effects and inconsistent	1.11	19
Common-effect and consistent → random-effects and inconsistent	2.01	75

This example shows that our methodology can be applied successfully to a very large network of treatments. The next example involves fewer treatments, and so, we use this to exhibit further aspects of our methodology.

### 5.2. Application two: smoking cessation

**Figure 2:**
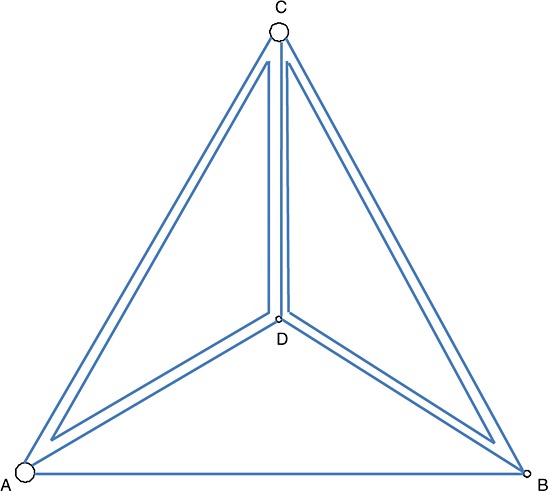
Network diagram for the smoking cessation data. Node size is proportional to the number of trials including the treatment, and multi-arm trials are shown as closed loops. Treatments are A: no contact, B: self-help, C: individual counselling and D: group counselling.

As a second example, we apply our methods to a dataset comparing interventions to aid smoking cessation. These data have been previously analysed by, amongst others, Lu and Ades 5 and Higgins *et al.* 7,  Table VI. The data consist of 24 trials comparing four treatments. There are two three-arm trials, giving a total of eight trial designs. The network diagram is shown in Figure 2. Here, binary data are available, where the outcome is smoking cessation, for which we use binomial distributions in an arm-based analysis where the treatment effects are log-odds ratios.

Results from fitting the model are given in Table 3. Again, we also report results with 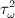 constrained to be zero, so assuming consistency. All interventions are estimated to be better than no contact in both cases, although posterior standard deviations are large. Allowing for inconsistency results in slightly different posterior means, with differences occurring at the first decimal place. The posterior mean for the inconsistency standard deviation *τ*_*ω*_ is moderately large but highly uncertain, suggesting there may be notable inconsistency within this network. Because it incorporates this extra degree of uncertainty, the model with inconsistency gives larger posterior standard deviations for treatment effects. However, conclusions regarding treatment rankings are similar, with group counselling being the preferred treatment in both cases.

**Table 3:** Results for the smoking cessation data.

		Consistency 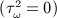		Inconsistency 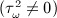	
	Parameter	Estimate (SD)	P(best)	Estimate (SD)	P(best)
Treatment					
A: No contact	–		0.00		0.00
B: Self-help	*δ*^*AB*^	0.49 (0.40)	0.06	0.60 (0.64)	0.09
C: Individual counselling	*δ*^*AC*^	0.84 (0.24)	0.24	0.95 (0.55)	0.25
D: Group counselling	*δ*^*AD*^	1.10 (0.44)	0.71	1.24 (0.67)	0.66
					
Heterogeneity mean (SD)	*τ*_*β*_	0.84 (0.19)		0.85 (0.20)	
median (CI)		0.82 (0.55, 1.27)		0.83 (0.55, 1.30)	
Inconsistency mean (SD)	*τ*_*ω*_			0.54 (0.51)	
median (CI)				0.40 (0.02, 1.90)	
					
DIC		326.60		326.69	

Two sets of results are shown, those assuming consistency and those allowing for inconsistency. Estimates are given by posterior means, and P(best) is the probability that each treatment is best (to two decimal places). Posterior medians and 95% credible intervals are also given for variance parameters.

SD, standard deviation; DIC, deviance information criterion.

Table 4 shows the *R* and *I*^2^ statistics for the smoking cessation data. The *I*^2^ statistic comparing the common-effect and consistent model to the random-effects and consistent model is high (90%), indicating that the between-study heterogeneity has considerable impact on the precision of the estimated treatment effects. The value of the *I*^2^ statistic comparing the random-effects model with inconsistency to this model assuming consistency is also high (63%), which means that the inconsistency variance also has considerable impact within this network. This is despite the fact that the DIC statistic for the random-effects consistency model is very slightly smaller than the one for the inconsistency model. Like Higgins *et al.*, 7, who instead use fixed effects for the inconsistency parameters, we conclude that there is not strong evidence of inconsistency in this network but it has quite considerable impact when it is included in the model. This is reminiscent of large *I*^2^ statistics but insignificant hypothesis tests for the presence of heterogeneity in conventional univariate random-effects meta-analyses. The average standard deviations (section 4) of the estimated treatment effects are 0.11 for the common-effect model assuming consistency, 0.33 for the random-effects model assuming consistency and 0.54 for the random-effects model with inconsistency.

**Table 4:** *R* and *I*^2^ statistics for the smoking cessation data.

	*R*	*I*^2^ (%)
Common-effect and consistent → random-effects and consistent	3.11	90
Random-effects and consistent → random-effects and inconsistent	1.63	63
Common-effect and consistent → random-effects and inconsistent	5.08	96

Estimates of the inconsistency parameters 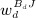 may shed light on the presence of inconsistency within this network. Posterior means and standard deviations of these inconsistency parameters are given in Table 5. The only inconsistency parameter that is estimated to be of a reasonable size is 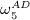, representing inconsistencies between trials including treatments *A* and *D* and other trial designs. However, there is just one trial that compares only treatments *A* and *D*, so the posterior standard deviation of 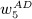 is larger than those for the other inconsistency parameters, and all the standard deviations are so large that it is difficult to draw any firm conclusions here.

**Table 5:** Estimated inconsistency parameters for the smoking cessation data.

Design	Parameter	Estimate (SD)
1: ACD	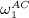	0.02 (0.56)
	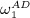	− 0.29 (0.67)
2: BCD	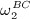	− 0.08 (0.56)
	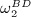	− 0.11 (0.57)
3: AB	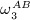	− 0.14 (0.57)
4: AC	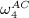	− 0.11 (0.52)
5: AD	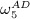	0.42 (0.82)
6: BC	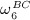	− 0.12 (0.57)
7: BD	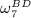	0.10 (0.58)
8: CD	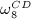	− 0.04 (0.52)

Estimates are given by posterior means.

SD, standard deviation.

The results for the basic parameters in Table 3 are in broad agreement with previous analyses of these data by Lu and Ades 5,  Table II and Dias *et al.* 9,  Table III. Qualitatively, we draw similar conclusions about the effectiveness of these four treatments to those who have previously used this dataset as an example.

#### 5.2.1. A sensitivity analysis

The inconsistency variance 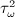 is likely to be poorly estimated when a network contains only a few inconsistency parameters, such as the smoking cessation network where the credible interval for *τ*_*ω*_ is very wide. In this case, we can conduct a sensitivity analysis to see how sensitive our conclusions are to different fixed values of *τ*_*ω*_. Table 6 gives results for the probability that each intervention is best when holding *τ*_*ω*_ fixed at various values. For the full model, the 95% credible interval for *τ*_*ω*_ was (0.02, 1.90), so values of *τ*_*ω*_ from 0 (i.e. assuming consistency) to 2 were considered. The probability of being the best treatment was distributed more evenly between treatments as inconsistency was increased, with the probability of group counselling being the best decreasing from 0.71 to 0.53 as the level of inconsistency increases. However, group counselling was still the treatment with the largest probability of being the best for all values of *τ*_*ω*_, which suggests that this conclusion is robust.

**Table 6:** Probability that each treatment is best for the smoking cessation data and various fixed values of the inconsistency standard deviation *τ*_*ω*_.

Treatment	*τ*_*ω*_				
	0	0.2	0.5	1	2
A: No contact	0.00	0.00	0.00	0.01	0.03
B: Self-help	0.06	0.06	0.08	0.13	0.19
C: Individual counselling	0.24	0.24	0.25	0.25	0.26
D: Group counselling	0.71	0.70	0.66	0.61	0.53

Estimates are given by posterior means.

## 6. Discussion

We have proposed a model for network meta-analysis that incorporates both between-study heterogeneity and inconsistency. We have shown how this is related to some of the models that have been previously proposed and have applied it to two real examples. We have based our approach on the design-by-interaction model, and extensions of our model are straightforward. For example, the design-by-treatment interaction model, of which our model is a special case, can easily incorporate study-level covariate effects, by adding these directly to model 3. If clinically relevant covariates are available to explain any apparent inconsistency in the network, for example, by substantially reducing the estimate of the inconsistency variance, then including them in the model could remove the need to model inconsistency. This is similar to the use of covariates to explain between-study variation in a meta-regression, and an investigation of which covariates appear to explain inconsistency in the network could be of interest in its own right. When there is insufficient evidence to demonstrate consistency or inconsistency, we let the analyst decide whether to include the extra source of variation in the same way that they currently need to decide whether to allow for heterogeneity using random effects in a univariate meta-analysis.

With sufficient replication within designs, richer models for the between-study heterogeneity involving more than a single parameter *τ*_*β*_ are certainly possible and should be considered in practice 33,34; ideally, a less structured between-study covariance matrix should be used, but this is only possible in situations where there are sufficient data available. For examples, with many different designs, richer models for the inconsistency parameters may be possible, and we leave it as an open question whether more sophisticated modelling of the network's inconsistency parameters should be attempted in applications that either facilitate or motivate this.

We have used WinBUGS to fit our model. Purpose built frequentist software, in order to avoid concerns related to prior sensitivity and convergence diagnostics and also to make our methods more accessible to applied researchers, would be a useful next step. However, the computation required for this is challenging because it involves relatively complex models, potentially high dimensional parameter spaces and variance components at two different levels of the model. Hence, at present, MCMC is probably the most convenient way to fit our model. The situations where the estimates of our two variance components are especially sensitive to their prior specification are also those where frequentist methods will struggle to provide accurate inference using conventional methods, but we hope that alternatives to our WinBUGS code will be developed and that this article will serve to encourage this.

We have estimated treatment effects and performed probabilistic ranking under the assumptions of our model and hence without assuming consistency. This might be considered controversial because it raises the obvious question of what it means to draw inferences when the available evidence is inconsistent. This question is perhaps related to the question of what the usual inferences from the conventional univariate random-effects model mean, where it is acknowledged that studies have different true underlying effects. Statisticians are now familiar with the interpretation of the estimated mean treatment effect from the random-effects model, but the issues are subtle 13,35 and the introduction of random inconsistency effects makes these yet more so. We interpret our estimates of treatment effect in a very similar way to conventional random-effects meta-analysis, where in our model, it is recognised that the study effects are different across both individual studies and their designs. Our probabilistic ranking also recognises this and ranks the studies on the basis of a statistical model that incorporates two different ways in which studies may have different underlying effects. Hence, our ranking may be both useful and meaningful and could be extended by calculating the probability that each treatment is second best, third best and so on. We could also rank the treatments in a new study, or a new design, using our model. However, efforts to explain the heterogeneity and inconsistency should be made in situations where the data are available to attempt this. We suggest that our model should be used to draw inferences only in reasonably large networks when small amounts of inconsistency are present that cannot be explained using covariates.

The entire model fit should be interpreted so that no single aspect of the model is used in isolation when making decisions. We suggest that, as a minimum, the estimates of the basic parameters and the variance components (with measures of their uncertainty), some form of ranking and the *I*^2^ and DIC (or another measure of model fit) statistics should be presented and interpreted when using our model. Further possibilities include the estimates of the inconsistency parameters and results from sensitivity analyses. By interpreting a variety of components of the model fit, and not just a single aspect, overly simplistic conclusions and specific misinterpretations should be avoided. Specifically, the type of misinterpretation that we wish to avoid would result from interpreting the estimated basic parameters whilst ignoring the rest of the fitted model. The basic parameters do not describe all important features of the data, such as the extent of the heterogeneity and the inconsistency, and so it is vital that the other types of results that we have presented are also used for inference. Further considerations, such as prediction intervals for different design types and shrunken estimates of the study effects 13, could also be used for this purpose. For example, prediction intervals for particular designs and treatment effects could be used to describe the effects in future studies, where these prediction intervals could relate either to designs already included in the network meta-analysis or to a new design.

The assumption of a normal distribution we make for the inconsistency parameters is quite strong, and we require a reasonably large number of design types to estimate the inconsistency variance with sufficient accuracy. In situations where these are not available, a sensitivity analysis can be used instead, but some may find this unsatisfactory because a sensitivity analysis does not provide a single answer. An alternative to a sensitivity analysis is to use an informative prior for the inconsistency variance. Instead, using fixed effects for the inconsistency parameters is a much weaker assumption that can be used regardless of the number of designs represented in the network. However, in addition to a coherent comparison of the multiple treatments, the probabilistic ranking of treatments is often an important aim of network meta-analysis, and this is more difficult to achieve when using fixed effects for the inconsistency parameters. We recognise that there are arguments for and against using fixed rather than random effects for the inconsistency parameters. Because neither approach is uniformly better, we suggest that both approaches have their merits and should both be considered in application. Random inconsistency effects are a useful device for handling inconsistent network meta-analyses and allow us to estimate average treatment effects across designs, but models using fixed effects for the inconsistency parameters are perhaps better for understanding where the inconsistencies in the network arise.

We have applied the *R* and *I*^2^ statistics for multivariate meta-analysis 14 to network meta-analysis. These are immediately applicable because they are based on generalised variances and our model uses random effects for both the between-study heterogeneity and the inconsistency. However, the development of alternative statistics that quantify the impact of between-study heterogeneity and inconsistency, derived more specifically for network meta-analysis, provides an avenue for further work.

## Supporting Information

Supporting info item
