# Supplementary material for: A design-by-treatment interaction model for network meta-analysis with random inconsistency effects
Source: Stat Med. 2014 Apr 29;33(21):3639–54. doi: 10.1002/sim.6188 (PMC4285290; doi:10.1002/sim.6188)
Supplement: Supplementary file 1 — Supporting info item [file sim0033-3639-sd1.pdf]

**Research Article**

Statistics in Medicine

Received XXXX

(www.interscience.wiley.com) DOI: 10.1002/sim.0000

# Supplementary information: A design-by-treatment interaction model for network meta-analysis with random inconsistency effects

## WinBUGS code for OAK application

The WinBUGS code we used for the analysis of the OAK data is given below (see section 5.1 for details). The code has been adapted from the code given in references [10, 23, 24] of the paper. Here i denotes the trial, i = 1,. . . ,87.

The data for trial i consist of t[i,1], ..., t[i,4], giving the set of treatments used (t[i,3] and t[i,4] may be NA if trial i includes fewer than four treatments), y[i,2], ..., y[i,4] giving the standardised mean differences for up to three treatment comparisons, var[i,2], ..., var[i,4] giving the variances of the standardised mean differences and cov[i] giving the within-study covariance. Also na[i] gives the number of arms of trial i, no[i] gives the number of observed standardised mean differences and des[i] gives the design of the trial, des[i]=1, ..., 38. In general na[i] = no[i] + 1, except for one trial which has two observed standardised mean differences comparing treatments A and H. Also included as data are nt, giving the number of treatments, ns2, ns3 and ns4, giving the numbers of two-arm, three-arm and four-arm trials respectively, ndes giving the number of designs, nades[] giving the number of arms in each design, and studies.dup which specifies the trial with two observations of the same treatment comparison.

The notation used in the code is similar to that used in the paper. In addition eta[i,k] is the fixed effect of the kth treatment of trial i relative to the baseline plus its random effect in trial i. Also om[des[i],k] is the inconsistency parameter for the kth treatment of the design of trial i.

```
model{

# STUDY 76 HAS TWO OBSERVATIONS OF ONE TREATMENT COMPARISON
# SET RANDOM EFFECT AND INCONSISTENCY TO BE THE SAME FOR BOTH OBSERVATIONS
eta[studies.dup,(no[studies.dup]+1)] <- eta[studies.dup,na[studies.dup]]
om[des[studies.dup],(no[studies.dup]+1)] <- om[des[studies.dup],na[studies.dup]]

# NORMAL LIKELIHOOD
# LOOP THROUGH 2-ARM STUDIES
for(i in 1:ns2){
    mu[i,2] <- eta[i,2] + om[des[i],2]
    prec[i] <- 1/var[i,2]
    y[i,2] ~ dnorm(mu[i,2],prec[i])
  }
# LOOP THROUGH 3-ARM STUDIES
for(i in (ns2+1):(ns2+ns3)){
    for(k in 1:no[i]){
        for(j in 1:no[i]){
# WITHIN-STUDY COVARIANCE
            Sigma[i,j,k] <- cov[i]*(1-equals(j,k))+var[i,k+1]*equals(j,k)
          }
# TRUE TREATMENT EFFECT IN STUDY i
        mu[i,k+1] <- eta[i,k+1]+om[des[i],k+1]
```

```
      }
# WITHIN-STUDY PRECISION
    Prec[i,1:no[i],1:no[i]] <- inverse(Sigma[i,,])
    y[i,2:(no[i]+1)] ~ dmnorm(mu[i,2:(no[i]+1)],Prec[i,1:no[i],1:no[i]])
  }
# LOOP THROUGH 4-ARM STUDIES
for(i in (ns2+ns3+1):(ns2+ns3+ns4)){
    for(k in 1:no[i]){
        for(j in 1:no[i]){
            Sigma2[i,j,k] <- cov[i]*(1-equals(j,k))+var[i,k+1]*equals(j,k)
          }
        mu[i,k+1] <- eta[i,k+1]+om[des[i],k+1]
      }
    Prec2[i,1:no[i],1:no[i]] <- inverse(Sigma2[i,,])
    y[i,2:(no[i]+1)] ~ dmnorm(mu[i,2:(no[i]+1)],Prec2[i,1:no[i],1:no[i]])
  }

# RANDOM EFFECTS DISTRIBUTION
for(i in 1:(ns2+ns3+ns4)){    # LOOP THROUGH ALL STUDIES
    w[i,1] <- 0
    eta[i,1] <- 0
    for(k in 2:na[i]){        # LOOP THROUGH ARMS
        eta[i,k] ~ dnorm(m.cond[i,k],precbeta.cond[i,k])
# MEANS WITH MULTI-ARM TRIAL CORRECTION
        m.cond[i,k] <-  delta[t[i,k]] - delta[t[i,1]] + sw[i,k]
# BETWEEN-STUDY PRECISION WITH MULTI-ARM TRIAL CORRECTION
        precbeta.cond[i,k] <- precbeta * 2 * (k-1) /k
        w[i,k] <- (eta[i,k] - delta[t[i,k]] + delta[t[i,1]])
        sw[i,k] <- sum(w[i,1:(k-1)])/(k-1)
      }
  }

# INCONSISTENCY PARAMETERS
for (i in 1:ndes)  {         # LOOP THROUGH DESIGNS
    om[i,1] <- 0
    for(k in 2:nades[i])  {   # LOOP THROUGH ARMS OF DESIGN i
        om[i,k] ~ dnorm(mom.cond[i,k],precom.cond[i,k])
# MEAN OF INCONSISTENCY DISTRIBUTION WITH MULTI-ARM TRIAL CORRECTION
        mom.cond[i,k] <- sum(om[i,1:k-1])/(k-1)
# PRECISION OF INCONSISTENCY DISTRIBUTION WITH MULTI-ARM TRIAL CORRECTION
        precom.cond[i,k] <- precomega * 2 * (k-1)/k
      }
  }

# TREATMENT EFFECT IS ZERO FOR REFERENCE TREATMENT
delta[1]<-0
# VAGUE PRIORS
for (k in 2:nt){  delta[k] ~ dnorm(0,.0001) }
taubeta ~ dunif(0,5)
precbeta <- pow(taubeta,-2)
tauomega ~ dunif(0,5)
precomega <- pow(tauomega,-2)

# PROBABILITIES OF BEING THE BEST TREATMENT
#(assuming high scores are bad). If high scores are good replace equals(rk[k],1) with equals(rk[k],nt)
for(k in 1:nt){
    rk[k] <- rank(delta[],k)
    best[k] <- equals(rk[k],1)
  }
```

```
}

DATA
list(nt=22,ns2=75,ns3=11,ns4=1,ndes=38,nades=c(2,2,2,2,2,2,2,2,2,2,2,2,2,2,2,2,2,2,2,2,2,2,2,2,2,2,3,3,
3,3,3,3,3,3,3,3,3,4),studies.dup=76)

t[,1] t[,2] y[,2] var[,2] t[,3] y[,3] var[,3] t[,4] y[,4] var[,4] cov[] na[] no[] des[]
2 20 0.107898285 0.023267397 NA NA NA NA NA NA NA 2 1 1
2 20 -0.181892962 0.048399075 NA NA NA NA NA NA NA 2 1 1
2 20 0.614138223 0.060716278 NA NA NA NA NA NA NA 2 1 1
2 20 -0.826036935 0.108779839 NA NA NA NA NA NA NA 2 1 1
1 6 -0.149161681 0.034305608 NA NA NA NA NA NA NA 2 1 2
1 22 -0.114197207 0.071545009 NA NA NA NA NA NA NA 2 1 3
2 5 -1.409772304 0.09614721 NA NA NA NA NA NA NA 2 1 4
2 5 -0.332409946 0.078099502 NA NA NA NA NA NA NA 2 1 4
2 5 0.068562705 0.091720085 NA NA NA NA NA NA NA 2 1 4
2 5 -0.364640272 0.081701784 NA NA NA NA NA NA NA 2 1 4
2 5 -0.751692275 0.155995944 NA NA NA NA NA NA NA 2 1 4
2 5 -0.503247648 0.082532582 NA NA NA NA NA NA NA 2 1 4
2 5 0.497196659 0.134697904 NA NA NA NA NA NA NA 2 1 4
2 22 -0.242640196 0.108982733 NA NA NA NA NA NA NA 2 1 5
2 22 -2.935180032 0.271692305 NA NA NA NA NA NA NA 2 1 5
2 22 -0.639754767 0.078410718 NA NA NA NA NA NA NA 2 1 5
2 22 -0.777482031 0.069083161 NA NA NA NA NA NA NA 2 1 5
22 9 0.061092357 0.100046653 NA NA NA NA NA NA NA 2 1 6
8 19 -1.192535248 0.094221403 NA NA NA NA NA NA NA 2 1 7
2 17 -0.645702769 0.102133115 NA NA NA NA NA NA NA 2 1 8
2 10 -0.841510096 0.145730892 NA NA NA NA NA NA NA 2 1 9
2 10 0.279216392 0.025958513 NA NA NA NA NA NA NA 2 1 9
2 10 0.149996775 0.06099994 NA NA NA NA NA NA NA 2 1 9
1 8 -0.197718631 0.059110975 NA NA NA NA NA NA NA 2 1 10
1 8 -0.469407176 0.077792994 NA NA NA NA NA NA NA 2 1 10
1 8 -0.956467054 0.061908536 NA NA NA NA NA NA NA 2 1 10
1 8 -0.393135753 0.023860206 NA NA NA NA NA NA NA 2 1 10
1 8 -0.20897436 0.0565442 NA NA NA NA NA NA NA 2 1 10
1 8 -0.578805813 0.198885666 NA NA NA NA NA NA NA 2 1 10
1 8 -0.020508176 0.043498863 NA NA NA NA NA NA NA 2 1 10
1 8 -0.067854499 0.200115106 NA NA NA NA NA NA NA 2 1 10
1 8 -0.444119732 0.011231151 NA NA NA NA NA NA NA 2 1 10
1 8 -0.437697528 0.146278199 NA NA NA NA NA NA NA 2 1 10
1 8 -0.659089443 0.076700404 NA NA NA NA NA NA NA 2 1 10
1 8 -0.458140438 0.078941276 NA NA NA NA NA NA NA 2 1 10
1 8 -0.438409877 0.033108781 NA NA NA NA NA NA NA 2 1 10
1 8 -0.207899048 0.041036847 NA NA NA NA NA NA NA 2 1 10
1 7 -0.513662422 0.04493278 NA NA NA NA NA NA NA 2 1 11
1 7 -0.41349784 0.136778246 NA NA NA NA NA NA NA 2 1 11
1 7 -4.287954289 0.541342151 NA NA NA NA NA NA NA 2 1 11
1 17 -0.074974082 0.066713509 NA NA NA NA NA NA NA 2 1 12
13 4 0.052107046 0.129210459 NA NA NA NA NA NA NA 2 1 13
13 4 -1.061520279 0.052069256 NA NA NA NA NA NA NA 2 1 13
13 4 -0.530687036 0.047054709 NA NA NA NA NA NA NA 2 1 13
13 4 -0.062733146 0.0727871 NA NA NA NA NA NA NA 2 1 13
13 4 -0.282867988 0.101000179 NA NA NA NA NA NA NA 2 1 13
13 4 -1.775793766 0.267377412 NA NA NA NA NA NA NA 2 1 13
13 4 -0.779492582 0.215190217 NA NA NA NA NA NA NA 2 1 13
1 4 -1.135560606 0.063636982 NA NA NA NA NA NA NA 2 1 14
1 4 -0.757081013 0.142886194 NA NA NA NA NA NA NA 2 1 14
1 4 0.166154589 0.100345092 NA NA NA NA NA NA NA 2 1 14
1 4 -1.698758855 0.202386435 NA NA NA NA NA NA NA 2 1 14
1 4 0.165518117 0.118049945 NA NA NA NA NA NA NA 2 1 14
```

```
1 4 -1.061592688 0.01334994 NA NA NA NA NA NA NA 2 1 14
2 19 -0.200997048 0.078620879 NA NA NA NA NA NA NA 2 1 15
2 19 -0.377311013 0.217675324 NA NA NA NA NA NA NA 2 1 15
2 19 -0.699644821 0.235819524 NA NA NA NA NA NA NA 2 1 15
2 16 0.351038827 0.076026902 NA NA NA NA NA NA NA 2 1 16
2 16 -1.215327548 0.10389677 NA NA NA NA NA NA NA 2 1 16
2 16 -0.07204235 0.100064876 NA NA NA NA NA NA NA 2 1 16
1 18 0.455570995 0.121107687 NA NA NA NA NA NA NA 2 1 17
3 8 -2.449678948 0.35594513 NA NA NA NA NA NA NA 2 1 18
3 8 -0.710669978 0.050616011 NA NA NA NA NA NA NA 2 1 18
1 11 -0.317478727 0.022278837 NA NA NA NA NA NA NA 2 1 19
1 11 -0.327130304 0.102365496 NA NA NA NA NA NA NA 2 1 19
1 11 -0.194728125 0.098548552 NA NA NA NA NA NA NA 2 1 19
1 12 -0.726290037 0.057776625 NA NA NA NA NA NA NA 2 1 20
1 12 0.089668992 0.010668453 NA NA NA NA NA NA NA 2 1 20
9 8 -0.802906052 0.086446581 NA NA NA NA NA NA NA 2 1 21
2 8 -0.634735456 0.11056433 NA NA NA NA NA NA NA 2 1 22
1 5 -1.011099738 0.056389517 NA NA NA NA NA NA NA 2 1 23
2 9 -0.201387548 0.078859156 NA NA NA NA NA NA NA 2 1 24
2 21 -0.822740296 0.103426504 NA NA NA NA NA NA NA 2 1 25
22 4 0.150803124 0.286526485 NA NA NA NA NA NA NA 2 1 26
2 15 -0.928262996 0.170416773 NA NA NA NA NA NA NA 2 1 27
1 8 -0.170139364 0.173748792 8 -0.035780873 0.176495692 NA NA NA 0.0588235294117647 2 2 10
1 4 -0.233571462 0.049754964 8 -0.025025514 0.033062697 NA NA NA 0.0163934426229508 3 2 28
3 22 -0.762857156 0.284852637 14 -0.433152233 0.263094018 NA NA NA 0.0833333333333333 3 2 29
1 4 -1.201925827 0.040793869 13 -0.684690514 0.045750321 NA NA NA 0.0149253731343284 3 2 30
1 4 -1.071267074 0.563756286 22 -1.313976021 0.595918499 NA NA NA 0.166666666666667 3 2 31
7 1 -0.053579736 0.037832677 12 -0.177808846 0.037324696 NA NA NA 0.0125 3 2 32
1 8 -0.496204998 0.251905638 17 -0.267065054 0.134522062 NA NA NA 0.0666666666666667 3 2 33
1 7 -0.439873163 0.024831019 8 -0.321458035 0.024362932 NA NA NA 0.0078740157480315 3 2 34
2 9 0 0.212669683 14 -0.689955396 0.231583482 NA NA NA 0.0769230769230769 3 2 35
3 15 -1.84964968 0.350038469 2 -0.19611917 0.412386578 NA NA NA 0.142857142857143 3 2 36
2 8 0.267938107 0.193227337 22 -0.171802395 0.188114918 NA NA NA 0.0625 3 2 37
2 4 -1.197750055 0.182785132 14 -0.260380447 0.16223164 22 -0.78114134 0.170387787 0.04 4 3 38
END
```

## WinBUGS code for smoking cessation application

The WinBUGS code we used for the analysis of the smoking cessation data is given below (see section 5.2 for details). The first section of the code specifies the binomial likelihood. The remainder of the code deals with the random effects and the inconsistency parameters and is identical to the equivalent part of the code used for the OAK data.

The data for trial `i` consist of `t[i,1]`, `t[i,2]`, `t[i,3]` giving the treatments included, `r[i,1]`, `r[i,2]`, `r[i,3]` giving the number of successes for each treatment and `n[i,1]`, `n[i,2]`, `n[i,3]` giving the number in each treatment arm. Again, `na[i]` gives the number of treatment arms in trial `i` and `des[i]` gives the design of trial `i`, `des[i]`=1, ..., 8. Also included as data are `nt`, giving the number of treatments, `ns` giving the number of trials , `ndes` giving the number of designs and `nades[]` giving the number of arms in each design. Here `base[i]` denotes the logit of the probability of success for the baseline treatment in trial `i`.

```
model{

# LOOP THROUGH STUDIES
for(i in 1:ns){

# BINOMIAL LIKELIHOOD WITH LOGIT LINK
# VAGUE PRIORS FOR TRIAL BASELINES
    base[i] ~ dnorm(0,.0001)
# LOOP THROUGH ARMS
    for (k in 1:na[i])  {
        r[i,k] ~ dbin(p[i,k],n[i,k])
        logit(p[i,k]) <- base[i] + eta[i,k] + om[des[i],k]
```

```
      }

# RANDOM EFFECTS DISTRIBUTION
w[i,1] <- 0
    eta[i,1]<-0
   for (k in 2:na[i]) {  # LOOP THROUGH ARMS
        eta[i,k] ~ dnorm(m.cond[i,k],precbeta.cond[i,k])
        m.cond[i,k] <-  delta[t[i,k]] - delta[t[i,1]] + sw[i,k]
        precbeta.cond[i,k] <- precbeta * 2 * (k-1)/k
        w[i,k] <- (eta[i,k] - delta[t[i,k]] + delta[t[i,1]])
        sw[i,k] <- sum(w[i,1:k-1])/(k-1)
      }
  }

# INCONSISTENCY PARAMETERS
for (i in 1:ndes)  {
    om[i,1] <- 0
    for(k in 2:nades[i])  {
        om[i,k] ~ dnorm(mom.cond[i,k],precom.cond[i,k])
        mom.cond[i,k] <- sum(om[i,1:k-1])/(k-1)
        precom.cond[i,k] <- precomega * 2 * (k-1)/k
      }
  }

delta[1]<-0
# VAGUE PRIORS
for (k in 2:nt){  delta[k] ~ dnorm(0,.0001) }
taubeta ~ dunif(0,5)
precbeta <- pow(taubeta,-2)
tauomega ~ dunif(0,5)
precomega <- pow(sdom,-2)

# PROBABILITIES OF BEING THE BEST TREATMENT
# (assuming events are good). If events are bad replace equals(rk[k],nt) with equals(rk[k],1)
  for(k in 1:nt){
    rk[k] <- rank(delta[],k)
    best[k] <- equals(rk[k],nt)
  }

}

DATA

list(nt=4,ns=24,ndes=8,nades=c(3,3,2,2,2,2,2,2) )

r[,1] n[,1] r[,2] n[,2] r[,3] n[,3] t[,1] t[,2] t[,3] na[] des[]
9 140 23 140 10 138 1 3 4 3 1 #  ACD
11 78 12 85 29 170 2 3 4 3 2 # BCD
79 702 77 694 NA 1 1 2 NA 2 3  # AB
18 671 21 535 NA 1 1 2 NA  2 3  # AB
8 116 19 149 NA 1 1 2 NA 2 3  # AB
75 731 363 714 NA 1 1 3 NA 2 4  # AC
2 106 9 205 NA 1 1 3 NA 2 4  # AC
58 549 237 1561 NA 1 1 3 NA 2 4 # AC
0 33 9 48 NA 1 1 3 NA 2 4  # AC
3 100 31 98 NA 1 1 3 NA 2 4 #  AC
1 31 26 95 NA 1 1 3 NA 2 4 # AC
6 39 17 77 NA 1 1 3 NA 2 4  # AC
64 642 107 761 NA 1 1 3 NA 2 4  # AC
5 62 8 90 NA 1 1 3 NA 2 4  # AC
```

```
20 234 34 237 NA 1 1 3 NA 2 4 # AC
95 1107 143 1031 NA 1 1 3 NA 2 4 # AC
15 187 36 504 NA 1 1 3 NA 2 4 # AC
78 584 73 675 NA 1 1 3 NA 2 4 # AC
69 1177 54 888 NA 1 1 3 NA 2 4 # AC
0 20 9 20 NA 1 1 4 NA 2 5 # AD
20 49 16 43 NA 1 2 3 NA 2 6 # BC
7 66 32 127 NA 1 2 4 NA 2 7 # BD
12 76 20 74 NA 1 3 4 NA 2 8 # CD
9 55 3 26 NA 1 3 4 NA 2 8 # CD
END
```

## Estimates of the inconsistency parameters for the OAK application

The reader should note that treatments were not always ordered alphabetically within designs for the OAK application. This has no consequences for the estimation but this different ordering of the treatments within designs is reflected in the subscripts corresponding to the inconsistency parameters.

**Table 1.** Estimated inconsistency parameters for the OAK data. Estimates are given by posterior means.

| Design | Parameter | Estimate (SD) |
|---|---|---|
| 1: BT | $\omega_1^{BT}$ | 0.00 (0.17) |
| 2: AF | $\omega_2^{AF}$ | 0.00 (0.17) |
| 3: AV | $\omega_3^{AV}$ | 0.05 (0.17) |
| 4: BE | $\omega_4^{BE}$ | 0.05 (0.17) |
| 5: BV | $\omega_5^{BV}$ | -0.07 (0.16) |
| 6: VI | $\omega_6^{VI}$ | -0.05 (0.17) |
| 7: HS | $\omega_7^{HS}$ | -0.07 (0.18) |
| 8: BQ | $\omega_8^{BQ}$ | -0.03 (0.17) |
| 9: BJ | $\omega_9^{BJ}$ | 0.00 (0.17) |
| 10: AH | $\omega_{10}^{AH}$ | -0.01 (0.13) |
| 11: AG | $\omega_{11}^{AG}$ | -0.09 (0.18) |
| 12: AQ | $\omega_{12}^{AQ}$ | 0.02 (0.16) |
| 13: MD | $\omega_{13}^{MD}$ | -0.03 (0.16) |
| 14: AD | $\omega_{14}^{AD}$ | 0.02 (0.14) |
| 15: BS | $\omega_{15}^{BS}$ | 0.07 (0.18) |
| 16: BP | $\omega_{16}^{BP}$ | 0.00 (0.17) |
| 17: AR | $\omega_{17}^{AR}$ | 0.00 (0.17) |
| 18: CH | $\omega_{18}^{CH}$ | -0.04 (0.17) |
| 19: AK | $\omega_{19}^{AK}$ | 0.00 (0.17) |
| 20: AL | $\omega_{20}^{AL}$ | 0.01 (0.16) |
| 21: IH | $\omega_{21}^{IH}$ | -0.04 (0.17) |
| 22: BH | $\omega_{22}^{BH}$ | -0.02 (0.16) |
| 23: AE | $\omega_{23}^{AE}$ | -0.05 (0.17) |
| 24: BI | $\omega_{24}^{BI}$ | -0.01 (0.16) |
| 25: BU | $\omega_{25}^{BU}$ | 0.00 (0.17) |
| 26: VD | $\omega_{26}^{VD}$ | 0.02 (0.16) |
| 27: BO | $\omega_{27}^{BO}$ | 0.02 (0.17) |
| 28: ADH | $\omega_{28}^{AD}$ | 0.06 (0.17) |
| | $\omega_{28}^{AH}$ | 0.04 (0.16) |
| 29: CVN | $\omega_{29}^{CV}$ | 0.02 (0.17) |
| | $\omega_{29}^{CN}$ | 0.02 (0.17) |
| 30: ALM | $\omega_{30}^{AL}$ | -0.05 (0.16) |
| | $\omega_{30}^{AM}$ | -0.04 (0.16) |
| 31: ADV | $\omega_{31}^{AD}$ | -0.01 (0.17) |
| | $\omega_{31}^{AV}$ | -0.03 (0.17) |
| 32: GAL | $\omega_{32}^{GA}$ | -0.07 (0.17) |
| | $\omega_{32}^{GL}$ | -0.04 (0.17) |
| 33: AHQ | $\omega_{33}^{AH}$ | -0.01 (0.16) |
| | $\omega_{33}^{AQ}$ | 0.00 (0.16) |
| 34: AGH | $\omega_{34}^{AG}$ | 0.01 (0.16) |
| | $\omega_{34}^{AH}$ | 0.00 (0.15) |
| 35: BIN | $\omega_{35}^{BI}$ | 0.00 (0.16) |
| | $\omega_{36}^{BN}$ | -0.02 (0.17) |
| 36: COB | $\omega_{36}^{CO}$ | -0.01 (0.17) |
| | $\omega_{36}^{CB}$ | 0.01 (0.17) |
| 37: BHV | $\omega_{37}^{BH}$ | 0.05 (0.17) |
| | $\omega_{37}^{BV}$ | 0.03 (0.16) |
| 38: BDNV | $\omega_{38}^{BD}$ | -0.03 (0.16) |
| | $\omega_{38}^{BN}$ | 0.00 (0.16) |
| | $\omega_{38}^{BV}$ | -0.01 (0.16) |
